# The Effect of Exercise Training on Body Composition, Insulin Resistance and High Sensitivity C-reactive Protein (Hs-CRP) in Women With Polycystic Ovary Syndrome: A Pilot Study From North India

**DOI:** 10.7759/cureus.23994

**Published:** 2022-04-09

**Authors:** Upasana Pandit, Meenakshi Singh, Rajesh Ranjan, Vikas Gupta

**Affiliations:** 1 Department of Obstetrics and Gynaecology, Hindalco Hospital, Renukoot, IND; 2 Department of Obstetrics and Gynaecology, Lady Hardinge Medical College, Delhi, IND; 3 Community Medicine, Noida International Institute of Medical Sciences, Noida, IND; 4 Community Medicine, Birsa Munda Government Medical College, Shahdol, IND

**Keywords:** aerobic exercise, crp, insulin resistance, obesity, pcos

## Abstract

Background

Polycystic ovary syndrome (PCOS) is defined as clinical or biochemical hyperandrogenism, oligo/amenorrhea, and polycystic ovaries with or without increased ovarian volume. The goal of this study was to assess the effect of a 20-week home-based aerobic exercise programme on body composition, insulin resistance, and hs-CRP levels in women with PCOS.

Methods

This 12-month prospective study included 60 female patients diagnosed with PCOS, aged 20 to 40 years. The participants were divided into 2 groups, one for the experiment and the other for the control. For each participant, the 12-hour fasting blood samples were taken on two occasions i.e., 24 hours before the first session and 48 hours after the last session to measure the fasting glucose, fasting insulin, and hs-CRP. The Statistical Package for Social Sciences (SPSS) was used to conduct the analysis, and an association was considered significant when the p-value was less than 0.05.

Results

In the present study, there was a dropout rate of 16.7% (5/30) in the study group and 23.3% (7/30) in the control group. The baseline characteristics were comparable (p>0.05) between the study group and the control group during the enrolment. The BMI (kg/m^2^) among the study group before the exercise programme was 22.8±1.8 and it was significantly reduced to 21.1±1.9 after the exercise programme (p<0.05). The HOMA-IR and hs-CRP (mg/L) levels among the study group before the exercise programme were 3.2±1.5 and 6.7±2.7 respectively, and these were significantly reduced to 1.9±1.6 and 4.2±1.3 respectively after the exercise programme (p<0.05). In contrast, paired T-test analysis showed no such significant difference (p<0.05) for all variables (Weight, BMI, Waist, Hip, fasting glucose, Fasting insulin, and hs-CRP) among the control group during the study period.

Conclusion

In a group of female PCOS patients, a 20-week home-based aerobic exercise programme reduced weight, BMI, HOMA-IR, and hs-CRP. Although more research on the effects of aerobic exercises in PCOS is needed, these findings support aerobic exercise's effectiveness in reducing inflammation and enhancing insulin sensitivity in these patients.

## Introduction

The most commonly occurring endocrinological condition among reproductive age group women is polycystic ovary syndrome (PCOS), which is characterized by clinical or biochemical hyperandrogenism, oligo/amenorrhea, and polycystic ovaries with or without increased ovarian volume [1]. Hyperandrogenism, characterized by hirsutism, persisting acne, or deranged biochemical profile, which includes raised androgen, the precursor of sex steroids, and metabolite of glucuronidated androgens (estrogens), is the most consistent and apparent feature. Worldwide, PCOS is affecting around 6.6% of reproductive-aged women, and in India, the prevalence of PCOS ranges between 2.2% to 26% among reproductive-aged women [2].

As such, there is no gold standard regarding management among females with PCOS who do not seek to get pregnant over the long term. Oral contraceptives, insulin sensitizers, and lifestyle modifications are among the treatments for hyperandrogenism and menstrual irregularities [3]. Despite a lack of evidence, oral contraceptive (combined low dose) are the preferred treatment of choice for controlling such symptoms over a longer duration of time [4]. Metformin, an insulin-sensitizing drug, has benefits among females having PCOS, although with no benefit in enhancing the ability to conceive. Most importantly, there is a necessity to look into the side effects while considering the beneficial effects of these treatments or drugs as adverse events including gastrointestinal (GI) and disturbed metabolism are well documented with such therapies [5].

The first line of treatment for metabolic problems in overweight and obese women with PCOS is lifestyle management, which focuses primarily on diet and physical activity and might help in enhancing the ovulation process, thus improving fertility as well. A low-carbohydrate diet paired with physical activity improves reproductive function [6]. In a study, aerobic physical exercise, compared to a hypocaloric high-protein diet, showed reduced levels of sex steroids, improved ovulation process and rates, and more regular menstrual cycles [7].

Some cytokines, such as Tumor Necrosis Factor-alpha (TNF-α), Interleukin-6 (IL-6), and high sensitivity C-Reactive Protein (hs-CRP), are associated with higher-than-normal levels of mild chronic inflammation [8]. Inflammatory biomarkers are shown to be elevated in PCOS patients, and there is a correlation between these indicators and infertility, as well as metabolic and cardiovascular concerns. This suggests that inflammation plays a key role in the etiology of insulin resistance in PCOS patients [9].

The majority of earlier research focused on the impact of exercise on lipid profiles and blood pressure, with only a few studies evaluating the effects of exercise on inflammation [10]. As a result, given the involvement of inflammation in PCOS pathogenesis and the findings of previous studies, more research is needed to determine the efficacy of exercise on inflammatory markers in these patients. The objective of this study was to assess the effect of a 20-week home-based aerobic exercise program on body composition, insulin resistance, and hs-CRP levels in women with polycystic ovarian syndrome.

## Materials and methods

Study setting and design

After receiving ethical approval from the Institutional Ethics Committee (IEC: Lady Harding Medical College, New Delhi; Approval Number: LHMC/IEC/122/2020; dated: 15/12/2020), the current quasi-experimental study was conducted for a period of 12 months (January 2021 to December 2021) in the Department of Obstetrics and Gynecology of a tertiary care teaching and referral hospital in Delhi, India.

Study subjects and sample size

The subjects in this study were female patients (20-40 years old) who had been diagnosed with PCOS using the Rotterdam criteria. To diagnose PCOS, two of three criteria must be present, namely oligo-and/or anovulation, clinical and/or biochemical manifestations of excess androgen, and polycystic ovaries found on ultrasound [11]. Females who were unable to do exercise, who had a history of cigarette smoking or alcohol consumption, who were taking any medication in the last 30 days that could alter laboratory test results (oral contraceptives, anti-diabetic drugs, anti-androgenic treatments, oocyte induction treatment, or any corticosteroid substance), who had other endocrine disorders such as Cushing syndrome, or had androgen-secreting tumors, or who were pregnant were excluded from the study. Before subjects were included in the study, written informed consent was obtained from them following a thorough explanation of the study's purpose, and a total of 60 patients were enrolled in the study during the first eight months. The subjects were categorized into two groups: experimental (aerobic exercise group, 30 patients) and control (no intervention, 30 patients).

Data collection and blood sample

Following enrolment in the study, a clinical history was recorded, and subject-specific and relevant information was gathered through interviews in a structured data collecting schedule. Fasting glucose, fasting insulin, and hs-CRP were measured twice (24 hours before the first session and 48 hours after the last session of the activity) via 12-hour fasting blood samples collected. A blood sample was collected at set hours of the day (8-10 a.m.) to ensure that diurnal variations in blood insulin and hs-CRP levels were not affected. Insulin levels were determined using the Sandwich ELISA method, and serum hs-CRP levels were determined using the immuno-turbidimetric method. To assess insulin resistance, the Homeostatic Model of Assessment of Insulin Resistance (HOMA-IR) was derived using fasting glucose and insulin levels [12].

Home-based aerobic exercise training

A daily aerobic exercise routine was followed by the experimental group. The physical exercise program included 20 weeks of consistent exercise (five sessions per week), which included brisk walking, cycling, or any other aerobic exercise at a self-selected pace described as faster than normal walking at a pace that could be sustained for at least 30 minutes at least three days per week [13]. A heart rate monitor (ECG2, Sports Instruments) was used to assure a heart rate of 120 beats per minute or above during physical activity. Subjects met with the principal investigator to discuss how to undertake their physical exercise and were then monitored by the principal investigator via weekly phone conversations to provide advice on how to continue their physical exercise. All of the exercises were done in addition to regular physical activity.

Anthropometric measurements

SECA Digital Weigh was used to weigh the subjects with 0.1 kg precision. The subject's height was measured with a 0.1 cm measurement tape attached to the wall. The BMI was computed by dividing the body in kg by height in meters squared. Using an anthropometric tape, the waist circumference was measured 2 cm above the highest lateral border of the iliac crest, and the hip circumference was measured around the widest portion of the buttocks.

Statistical analysis

The data was imported into a Microsoft Excel spreadsheet and analyzed with the Statistical Package for Social Sciences (SPSS) version 26. Each group of study patients' baseline demographic, clinical, and laboratory data was used to analyze the results. Continuous variables were reported as mean ± SD, whereas categorical variables were presented as number and percentage (%). The Kolmogorov-Smirnov test was used to determine the data normality. The non-parametric test was employed if the normality was refused. The mean of baseline demographic variables, laboratory parameters, and anthropometric measurements was compared across the groups using an unpaired t-test, and within groups using a Paired t-test. All tests were run at a 5% level of significance; an association was considered significant if the p-value was < 0.05.

## Results

In the present study, a total of 60 subjects (30 each in the study and control group) were enrolled, but there was a dropout rate of 16.7% (5/30) in the study group and 23.3% (7/30) in the control group. The comparison of baseline characteristics showed that all study variables were comparable (p>0.05) between the study group and control group during the enrolment which allowed analysis to not be affected due to selection bias (Table 1). 

**Table 1:** Baseline characteristics comparison (preintervention) of study and control groups (N=48). # BMI= Body Mass Index, $ HOMA-IR= Homeostatic Model of Assessment of Insulin Resistance, * hs-CRP= high sensitivity C-Reactive Protein

Variables	Study group (n=25)	Control group (n=23)	p-value
Age (in years)	33.5+6.2	32.7+6.7	0.669
Weight (kg)	67.4+4.9	66.2+6.4	0.467
BMI (kg/m^2^)^#^	22.8+1.8	22.6+2.1	0.819
Waist (cm)	96.5+10.2	97.2+10.9	0.781
Hip (cm)	109.3+8.2	109.4+17.7	0.979
Fasting glucose (mmol/L)	4.8+0.7	5.1+0.9	0.201
Fasting insulin (μIU/mL)	14.7+8.4	13.9+8.7	0.747
HOMA-IR^$^	3.2+1.5	3.1+1.1	0.794
hs-CRP (mg/L)*	6.7+2.7	7.1+2.8	0.616

The paired T-test analysis showed that there was a significant difference (p<0.05) for all variables (weight, BMI, waist, hip, fasting glucose, fasting insulin, and hs-CRP) in the study group (preintervention and postintervention). The average weight (kg) of subjects of the study group prior to the exercise programme was 67.4±4.9 whereas it was reduced to 62.7±5.1 after such intervention. Similarly, the BMI (kg/m2) among the study group before the exercise programme was 22.8±1.8 and it was significantly reduced to 21.1±1.9 after the exercise programme (p<0.05). The HOMA-IR and hs-CRP (mg/L) levels among the study group before the exercise programme were 3.2±1.5 and 6.7±2.7 respectively, and these were significantly reduced to 1.9±1.6 and 4.2±1.3 respectively after the exercise programme (p<0.05). In contrast, paired T-test analysis showed no such significant difference (p<0.05) for all variables (Weight, BMI, Waist, Hip, fasting glucose, Fasting insulin, and hs-CRP) among the control group during the study period. The average weight (kg) of subjects in the control group at the start of the study was 66.2±6.4, whereas it was 66.1±4.7 at the end of the study. Similarly, BMI (kg/m2) among the control group was 22.6±2.1, and it was 22.9±2.7 after the end of the study. The HOMA-IR and hs-CRP (mg/L) levels among the control group were 3.1±1.1 and 7.1±2.8 respectively, and these were 3.3±1.4 and 7.2±2.4 respectively at the end of the study (Table 2).

**Table 2:** Comparison of anthropometric indices, hs-CRP, and insulin resistance index (preintervention vs postintervention) in study and control groups (N=48). # BMI= Body Mass Index, $ HOMA-IR= Homeostatic Model of Assessment of Insulin Resistance, * hs-CRP= high sensitivity C-Reactive Protein

Variables	Study group (n=25)		p-value	Control group (n=23)		p-value
	Baseline	After 20 weeks		Baseline	After 20 weeks	
Weight (kg)	67.4+4.9	62.7+5.1	0.001	66.2+6.4	66.1+4.7	0.958
BMI (kg/m^2^)^#^	22.8+1.8	21.1+1.9	0.002	22.6+2.1	22.9+2.7	0.676
Waist (cm)	96.5+10.2	90.2+9.7	0.029	97.7+15.6	96.9+13.1	0.851
Hip (cm)	109.3+8.2	103.2+9.9	0.021	109.4+17.7	110.1+12.8	0.878
Fasting glucose (mmol/L)	4.8+0.7	3.9+1.1	0.001	5.1+0.9	4.9+0.8	0.430
Fasting insulin (μIU/mL)	14.7+8.4	10.3+6.6	0.044	13.9+8.7	14.2+6.2	0.893
HOMA-IR^$^	3.2+1.5	1.9+1.6	0.004	3.1+1.1	3.3+1.4	0.592
hs-CRP (mg/L)*	6.7+2.7	4.2+1.3	0.001	7.1+2.8	7.2+2.4	1.000

The weight (kg) of the study group and control groups at the end of the study were 62.7+5.1 and 66.1+4.7 respectively and the BMI (kg/m2) at the end of the study was 21.1+1.9 and 22.9+2.7 among study and control groups respectively. The fasting glucose (mmol/L) levels among the study and control groups at the end of the study were 3.9+1.1 and 4.9+0.8 respectively. Similarly, fasting insulin (μIU/mL) levels among the study and control groups at the end of the study were 10.3+6.6 and 14.2+6.2 respectively. The comparison of baseline characteristics showed that all study variables were having statistically significant differences (p<0.05) between the study group and the control group at the end of the study period (Table 3). 

**Table 3:** Comparison of anthropometric indices, hs-CRP, and insulin resistance index (postintervention) for study and control groups (N=48). # BMI= Body Mass Index, $ HOMA-IR= Homeostatic Model of Assessment of Insulin Resistance, * hs-CRP= high sensitivity C-Reactive Protein

Variables	Study group (n=25)	Control group (n=23)	p-value
Weight (kg)	62.7+5.1	66.1+4.7	0.020
BMI (kg/m^2^)^#^	21.1+1.9	22.9+2.7	0.010
Waist (cm)	90.2+9.7	96.9+13.1	0.048
Hip (cm)	103.2+9.9	110.1+12.8	0.041
Fasting glucose (mmol/L)	3.9+1.1	4.9+0.8	0.001
Fasting insulin (μIU/mL)	10.3+6.6	14.2+6.2	0.040
HOMA-IR^$^	1.9+1.6	3.3+1.4	0.002
hs-CRP (mg/L)*	4.2+1.3	7.2+2.4	< 0.0001

## Discussion

In the present study, when compared to the control group, women with PCOS saw significant weight loss, improvements in insulin resistance, and hs-CRP levels following a home-based exercise program. Weight loss after exercise has been proven in several studies to improve body composition in women with PCOS by reducing waist circumference [14,15]. As it maintains resting metabolic rate and aids in sustained weight (i.e. adipose tissue) loss and/or weight maintenance, better preservation of waist circumference has crucial potential implications for long-term weight loss and maintenance [16]. Improved insulin sensitivity has been associated with a decrease in abdominal adipose tissue [17]. The effects of central body fat on insulin sensitivity and the ovaries as a result of hyperinsulinemia may indirectly contribute to hyperandrogenemia [18].

Insulin resistance was significantly reduced after the exercise program in this study. Using the homeostatic model of assessment (HOMA), the effect of exercise on insulin sensitivity was observed in several studies, finding a significant decrease in insulin resistance in the exercise group, [19,20], and few studies finding no significant change in HOMA-IR before and after exercise and studies [21,22]. However, these studies reported high serum levels of IGFBP-1 which have been proposed to be a sensitive marker of insulin sensitivity and this was supported by a significant negative correlation between IGFBP-1 and HOMA index after intervention (r2 = −0.48; P<.01). Aerobic exercise improves glucose elimination by increasing skeletal muscle capillarization, blood flow, and hexokinase and glycogen synthase activities, while exercise improves insulin sensitivity by increasing muscle mass and the amount of glucose transporter proteins [23].

Through its effect on body composition, exercise may help to combat the etiology of PCOS [24]. In type 2 diabetes, improvements in skeletal muscle size and quality have been accompanied by reductions in visceral fat and improvements in insulin sensitivity and glucoregulation as a result of progressive resistance training (PRT) [25]. Increased insulin sensitivity and glucoregulation, in turn, may lower androgen synthesis and hyperandrogenemia in women with PCOS, potentially halting the disease process (premature follicle growth arrest) and menstrual irregularity [24].

The group analysis in this study revealed that the exercise group decreased fasting glucose over time (p = 0.001). Previous aerobic-prescribed trials in PCOS, type 2 diabetes, and/or obesity have shown significant reductions in fasting insulin and fasting glucose [25,26].

The current study found an inverse association between regular physical activity and hs-CRP levels in the blood. The effects of exercise training on CRP varied depending on the type of exercise, and values were significantly lower than the control subjects in the previous studies [27]. After adjusting for confounding factors, bivariate analysis revealed that joggers (OR:0.33) and aerobic dancers (OR: 0.31) were considerably less likely than cyclists (OR: 1.30), swimmers (OR: 0.62), and weightlifters (OR: 0.83) to have increased CRP [28]. Other cross-sectional studies have found a similar negative relationship between physical activity and CRP [29,30].

In small-scale research, these physiological data are challenging to interpret. These adaptations will need to be investigated further in the future, including the assessment of dose-response effects for each outcome. More sensitive insulin resistance tests, such as the euglycaemic-hyperinsulaemic clamp, would be beneficial in this research.

Limitations

There were some limitations to this study. The trial period was not long enough to determine whether aerobic exercise had a long-term benefit. The authors were unable to compare different workout programmes using other ways of physical activity (resistance training, mixed aerobic, and resistance exercise). The current authors did not examine the patients' cholesterol changes. Finally, the women were not assigned to experimental or control groups using randomization.

## Conclusions

The present study showed that in a group of female PCOS patients, a 20-week home-based aerobic exercise programme reduced HOMA-IR and hs-CRP. Although more research on the effects of aerobic exercises on PCOS is needed, these findings support the effectiveness of aerobic exercise in reducing inflammation and enhancing insulin sensitivity in these patients. As per the present study findings, the patients with PCOS should be engaged in aerobic activity for effective outcome. Future research should look into the effect of aerobic exercises in PCOS patients with diabetes mellitus to see whether improving insulin resistance has similar effects on blood glucose levels.
